# SPH–FEM Analysis of Effect of Flow Impingement of Ultrasonic Honing Cavitation Microjet on Titanium–Tantalum Alloy Surface

**DOI:** 10.3390/mi15010038

**Published:** 2023-12-23

**Authors:** Jinwei Zhang, Xijing Zhu, Jing Li

**Affiliations:** 1 School of Mechanical Engineering, North University of China, Taiyuan 030051, China; s202102022@st.nuc.edu.cn (J.Z.); li-jing@nuc.edu.cn (J.L.); 2 Shanxi Provincial Key Laboratory of Advanced Manufacturing Technology, North University of China, Taiyuan 030051, China

**Keywords:** microjet impact, smooth particle fluid dynamics, ultrasonic honing, acoustic cavitation

## Abstract

To investigate the machining effect of ultrasonic honing microjets on a titanium–tantalum alloy surface, a cavitation microjet flow impingement model was established using the smoothed particle hydrodynamics–finite element method (SPH–FEM) coupling method including the effects of wall elastic–plastic deformation, the ultrasonic field and the honing pressure field. Simulation analysis was conducted on a single impact with different initial speeds and a continuous impact at a constant initial speed. The results showed that the initial speed of the microjet needed to reach at least 580 to 610 m/s in order to obtain an obvious effect of the single impact. The single impact had almost no effect at low speeds. However, when the microjet continuously impacted the same position, obvious pits were produced via a cumulative effect. These pits were similar to that obtained by the single impact, and they had the maximum depth at the edge rather than the center. With the increase in the microjet’s initial speed, the total number of shocks required to reach the same depth gradually decreases. When the number of impacts is large, with the increase in the number of impacts, the growth rate of the maximum pit depth gradually slows down, and even shows no growth or negative growth at some times. Using the continuous impacts of the microjet by prolonging the processing time can enhance titanium–tantalum alloy machining with ultrasonic honing for material removal.

## 1. Introduction

Using alloy materials or biological materials as implants to fill damaged or missing parts of bone tissues has been a common effective approach in medical practice. Since the surface of natural bone tissues is covered by multi-scale composite textures and holes at the micron, submicron and nano scales, the implant surface has important effects on cell behavior. For example, a micron-scale structure (1 to 100 μm) on the implant surface can provide helpful signals for cell adhesion and increase the contact area of cell pseudopodial adhesion to enhance the mechanical insertion force with bone tissues and regulate the migration and growth of bone cells. Among the various types of alloys, titanium–tantalum alloys have good biocompatibility and hence can be used as a good substitute for bone implantation. They have great potential in future biomedical applications [1,2,3,4].

During the machining process of titanium–tantalum alloys using ultrasonic vibration, the cavity collapse that occurs near the wall of the alloy component is complex. The free surface of the cavity far away from the wall shrinks faster than that close to the wall. The cavity’s side which is further from the wall moves toward the wall and finally runs through itself. A high-speed microjet is generated to impact the wall to produce a micro-cutting action and obtain a micron-level structure on the component surface. The traditional finite element method (FEM) has a problem of mesh distortion when it is used to handle large deformation problems, leading to inaccurate or difficult computation. The smoothed particle hydrodynamics (SPH) approach is a completely Lagrangian meshless particle method. It uses a set of particles to replace the elements in the FEM to discretize and approximate the governing equations of integrals or differentials. It can avoid the computational difficulties caused by meshes, and hence it has significant advantages in simulating large deformations, transient impact explosions and mesh distortion. However, it is not as accurate as the FEM in handling small deformation problems [5].

To retain the advantages of these two methods, an SPH particle model and a FEM model were established and coupled to create an SPH–FEM method. This method was used to simulate the impact process of cavitation microjets on the surface of a titanium–tantalum alloy during ultrasonic honing. Because the material hardness was high, it was difficult to simulate the effect of a single impact. Therefore, continuous impacts at one position with different initial microjet speeds were simulated. The morphology of the impact pit was analyzed statistically. The simulation results elucidate the impact mechanisms of ultrasonic cavitation microjets.

## 2. Theoretical Model

### 2.1. Mathematical Model of Fluid–Structure Coupling for Microjet Impact

Microjet impact on the wall surface is a kind of nonlinear fluid–structure coupling problem, which can be briefly regarded as a beam of high-speed water jet impact on the wall surface. The process can be seen to have two typical stages: the water hammer pressure stage and the stagnation pressure stage [6,7,8].

The instantaneous speed of the microjet decreases sharply when impacting the wall surface. At the same time, a shock wave is generated and propagates towards the liquid and the wall, respectively. The shock wave divides the liquid region into disturbed and undisturbed areas. In the disturbed area, before the shock wave leaves, the area will exhibit extremely high pressure due to an instantaneous reduction in the speed of the microjet. The compressibility of the liquid needs to be considered. This phase only lasts a very short time, which is the water hammer pressure phase.

Subsequently, the shock wave leaves the area. Because the pressure in the liquid in the disturbed area is much larger than the external atmospheric pressure, the liquid will be ejected at a high speed under pressure and form a high-speed lateral jet along the wall, the speed of which is greater than the initial speed of the microjet. After that, the pressure in the disturbed region decreases to a stable stagnation pressure and lasts for a relatively long time, which is the stagnation pressure stage.

**Figure 1:**
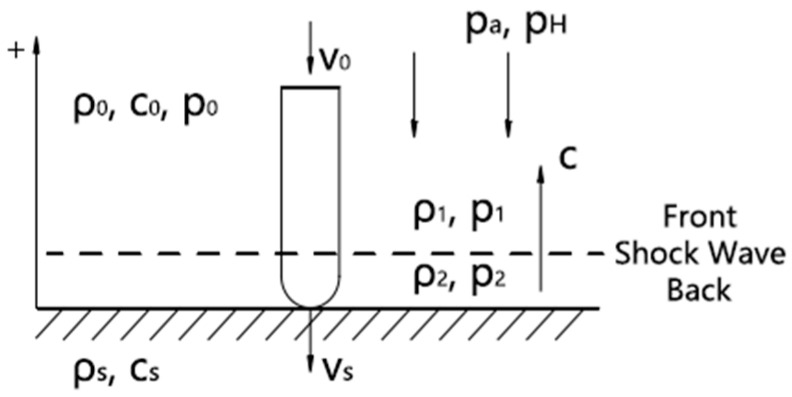
Schematic diagram of microjet impact.

Our research group has conducted mathematical modeling of the impact process of a microjet [8]. The model is shown in Figure 1. $\rho_{s}$ is the solid density, $c_{s}$ is the speed of sound in the solid, $\rho_{0}$ is the liquid density, $c_{0}$ is the speed of sound in the liquid, $p_{0}$ is the liquid pressure, $p_{a}=p_{A}sin\omega t$ is the ultrasonic pressure, $p_{A}$ is the amplitude of ultrasonic pressure and $p_{H}$ is the honing tool pressure. We use the stationary wall as a reference frame, where $v_{0}$ is the microjet’s initial speed.

Without considering the time effect, we can obtain a simple mathematical model. The water hammer pressure $p_{wh}$ and stagnation pressure $p_{st}$ values are given as follows:

(1) $$\left\{\begin{array}{c} p_{wh}=\rho_{0}c_{0}v_{0}\frac{1+kM_{0}}{1+\Gamma (1+kM_{0})}-\frac{\Gamma (1+kM_{0})(p_{a}+p_{H})}{1+\Gamma (1+kM_{0})}+p_{a}+p_{H}\\ p_{st}=\frac{1}{2}\rho_{0}{v_{0}}^{2}+p_{a}+p_{H} \end{array}\right.$$

where $\Gamma =(\rho_{0}c_{0})/(\rho_{s}c_{s})$, $M_{0}=v_{0}/c_{0}$ [9]. k is a liquid related constant and for water it is two.

If considering the time effect, we can obtain a three-dimensional mathematical model of the fluid–structure coupling of the microjet’s impact. The liquid’s formulas are given as follows:

(2) $$\left\{\begin{array}{c} \frac{\partial^{2}\Phi }{\partial t^{2}}-c^{2}\nabla^{2}\Phi =0\\ \Phi {|}_{t=0}=0,\;\;\nabla \Phi {|}_{{|}_{t=0}}=0\\ \frac{\partial \Phi }{\partial z}{|}_{z=0}=v_{0}-\frac{\partial u_{z}}{\partial t}{|}_{z=0} \end{array}\right.$$

The solid’s formulas are given as follows:

(3) $$\left\{\begin{array}{c} \rho_{s}\frac{\partial^{2}U}{\partial t^{2}}+\nabla \cdot \sigma =0\\ U{|}_{t=0}=0,\;\;\frac{\partial U}{\partial t}{|}_{t=0}=(0,\;0,\;v_{0})\\ \partial z{|}_{z=0}=p_{2}{|}_{z=0} \end{array}\right.$$

where $\nabla =\frac{\partial }{\partial x}i+\frac{\partial }{\partial y}j+\frac{\partial }{\partial z}k$**,** ∇ is the divergence, Φ is the velocity potential function, $U(u_{x},u_{y},u_{z})$ is the wall particle displacement and σ is the particle stress tensor.

The mathematical model is a system of partial differential equations and obtaining its analytical solution is very difficult. Therefore, finite element simulation is used to analyze it.

### 2.2. Constitutive Model Selection of Metal Material

Combined with the microjet impact mathematical model, during the impact process, the wall surface exhibits characteristics of instantaneous strong dynamic load, large deformation and high strain rate. The strain rate in the deformation process of materials can be as high as 10^4^ to 10^6^ s^−1^ and the strain rate effect cannot be ignored. Therefore, the classical Johnson–Cook model is chosen to describe the mechanical behavior changes in materials. The common formula of the J–C model gives the expression of yield stress $\sigma_{e}$ as follows [10]:

(4) $$\sigma_{e}=(\sigma_{0}+B{\varepsilon_{e}}^{n1})(1+Cln(\dot{\varepsilon_{e}}/\dot{\varepsilon_{0}}))\left\{1-{\left(\frac{T-T_{room}}{T_{melt}-T_{room}}\right)}^{m}\right\}$$

where $\sigma_{0}$ is the yield strength, $\varepsilon_{e}$ is the equivalent strain, $\dot{\varepsilon_{e}}$ is the equivalent strain rate, $\dot{\varepsilon_{0}}$ is the reference strain rate, *B* and *n*1 are strain hardening parameters, *C* is the strain rate strengthening parameter, *T* is the temperature during material deformation, $T_{room}$ is the room temperature, $T_{melt}$ is the material melting point and *m* is the temperature sensitive parameter.

The elastic deformation σ is considered to be described by Hooke’s law, which is independent of strain rate, as follows:

(5) σ=Eε=2G(1+P)ε

where *E* is the Young’s modulus, *G* is the shear modulus and *P* is the Poisson’s ratio. The parameters are treated as a constant and taken as the value at room temperature and pressure.

### 2.3. Basic Theory of SPH

The SPH method is particle-based, and it considers the fact that all physical quantities are carried by particles. The governing partial differential equations can be approximated using the SPH method with two steps: kernel approximation and particle approximation [11].

Kernel approximation is the integration of smooth kernel function. Particle approximation is the superposition summation of all particles’ values in a finite region. In the SPH theory, the kernel approximation of any continuous function *f(x)* in a variable field Ω is given as follows [11]:

(6) $$<f\left(x\right)>=\int_{\Omega }^{\;}f\left(x^{\prime }\right)W(x-x^{\prime },\;h)dx\prime$$

where *h* is the smooth length, which defines the affected region of the smooth kernel function $W(x-x^{\prime },\;h)$. The selection of the smooth kernel function is important because the computational accuracy depends on the smooth kernel function. The widely used cubic B-spline smooth kernel function was selected in this study.

The SPH particle region was liquid in this study. Its Navier–Stokes equation can be discretized via the SPH method as follows [12]:

(7) $$\frac{d\rho_{i}}{dt}=\sum_{j=1}^{N}m_{j}({v_{i}}^{\beta }-{v_{j}}^{\beta })\frac{\partial W_{ij}}{\partial {x_{i}}^{\beta }}$$

(8) $$\frac{d{v_{i}}^{\alpha }}{dt}=\sum_{j=1}^{N}m_{j}(\frac{{\sigma_{i}}^{\alpha \beta }}{{p_{i}}^{2}}+\frac{{\sigma_{j}}^{\alpha \beta }}{{p_{j}}^{2}})\frac{\partial W_{ij}}{\partial {x_{i}}^{\beta }}$$

(9) $$\frac{de_{i}}{dt}=\frac{1}{2}\sum_{j=1}^{N}m_{j}\left(\frac{p_{i}}{{\rho_{i}}^{2}}+\frac{p_{j}}{{\rho_{j}}^{2}}\right){v_{ij}}^{\beta }\frac{\partial W_{ij}}{\partial {x_{i}}^{\beta }}+\frac{\mu_{i}}{2\rho_{i}}{\varepsilon_{i}}^{\alpha \beta }{\varepsilon_{j}}^{\alpha \beta }$$

The above three equations are the conservations of mass, momentum and energy, respectively. In these equations, $\rho_{i}$ is the particle density, $m_{j}$ is the mass of particle *j*, $x_{i}$ is the coordinate of particle *i* in the direction of *β* and $p_{i}$ is the isotropic pressure of particle *i*. The parameters $\sigma_{i}$ and $\varepsilon_{i}$ are the stress and strain tensors of particle *i*, respectively, $v_{ij}$ is the component of the relative speed between two particles in the direction of *β* and *μ* is the fluid viscosity coefficient. *N* is the total number of particles in the smooth length range. Moreover, $W_{ij}=W(x_{i}-x_{j},h)$.

## 3. Mass Fraction Selection of Titanium–Tantalum Alloy

**Figure 2:**
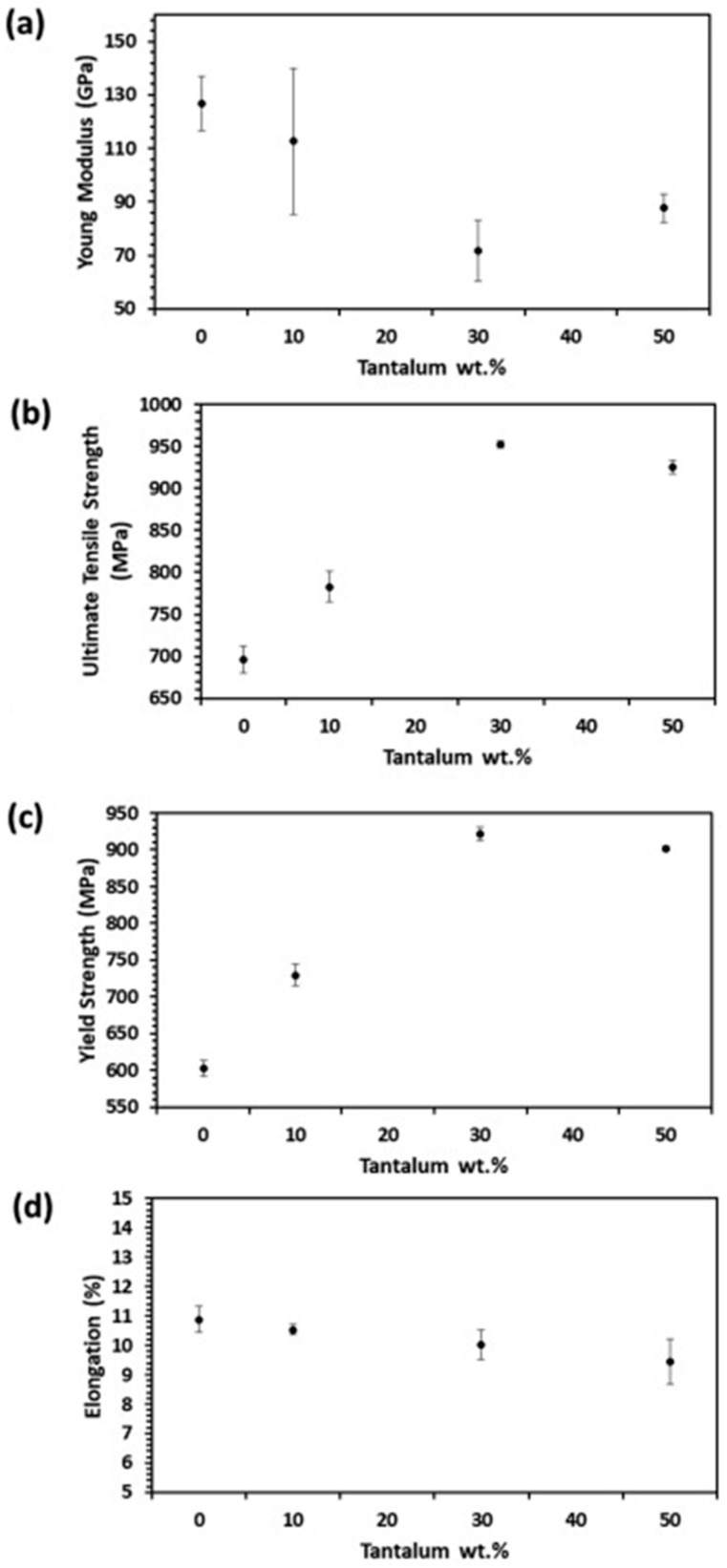
Mechanical properties of Ti–Ta alloys with different tantalum contents: (**a**) Young’s modulus; (**b**) ultimate tensile strength; (**c**) yield strength; and (**d**) elongation rate.

Previous researchers conducted tensile tests and Vickers hardness tests to reveal the mechanical properties of titanium–tantalum alloys with different tantalum contents, as shown in Figure 2 [13].

They found that the modulus of Ti30Ta (mass fraction: 70% Ti and 30% Ta) is the lowest, and its tensile strength and yield strength are the highest. Such mechanical properties are favorable for orthopedic applications. Therefore, Ti30Ta was selected as the wall material in this study.

## 4. Coupled Model of SPH–FEM

### 4.1. Model Building

**Figure 3:**
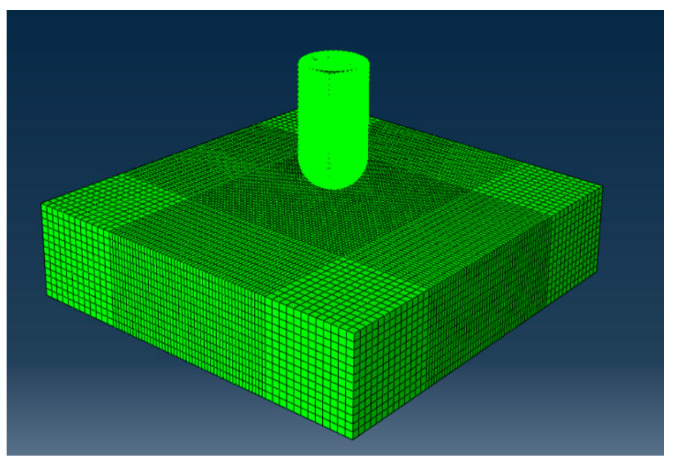
SPH–FEM coupled model of microjet impact.

The impact process involves large deformations of the microjets and small elastoplastic deformations of the alloy material [14]. The commercial finite element analysis software ABAQUS 2022 was selected in the SPH–FEM to build the model. The microjet impact model is shown in Figure 3.

The microjet was modelled by SPH particles. The keywords “* Section Controls” were used to control the parameters related to SPH, including artificial viscosity terms, smooth length, smooth function type and SPH region.

The component wall was divided into meshes via the FEM. The keywords “* Contact Inclusions” were used to establish the contact relationship between the SPH particle model and the FEM to conduct coupled analysis.

### 4.2. Model Parameters

According to the specific operating conditions of ultrasonic honing, the fluid viscosity, compressibility, surface tension and thermal effect between the media were ignored in the established model. The elastic–plastic deformation of the wall, the ultrasonic field and the honing pressure field were included in the model.

The honing fluid is usually water, kerosene or emulsion. Water is usually selected for rough honing, so we used it for fluid material. According to the range of the wall surface roughness during processing, the friction coefficient between the honing fluid and the wall was assumed to be 0.1. The diameter of the cavitation microjet was several microns [15]. The model parameters included the following: microjet diameter 5 μm, microjet length 12 μm and vertical impact to the wall. The microjet mesh type was PC3D. The total number of particles was 5982. The wall material was assumed to be Ti30Ta with a length of 40 μm, width of 40 μm and height of 10 μm. The wall mesh type was C3D8R. The mesh density was increased in the central impact area. There were 32,500 encrypted and 42,588 non-encrypted cells around the central area.

The fluid flow motion was controlled in the model using the equation of state $U_{s}-U_{p}$, $U_{s}=c_{0}+sU_{p}$, where $c_{0}$ and *s* are fluid sound velocity and a dimensionless parameter, respectively. This equation defines the linear relationship between the impact velocity $U_{s}$ and the particle velocity $U_{p}$, and *s =* 1.75 [16]. An elastoplastic material was selected for the solid part and the part was fixed along its perimeter. An ultrasonic pressure $p_{a}$ and a honing pressure $p_{H}$ of a sinusoidal function with periodic changes were applied in the model. The parameter $p_{H}$ was fixed at 0.24 MPa [17]. The parameter $p_{a}$ was assumed to be $p_{a}=P_{A}sin\omega t$, where ω=2πf, the sound pressure amplitude $P_{A}$ = 1.753 MPa and the ultrasonic frequency *f* = 20 kHz. The material parameters are shown in Table 1.

## 5. SPH–FEM Analysis of Microjet Impact on Titanium–Tantalum Alloy

### 5.1. Preliminary Analysis

**Figure 4:**
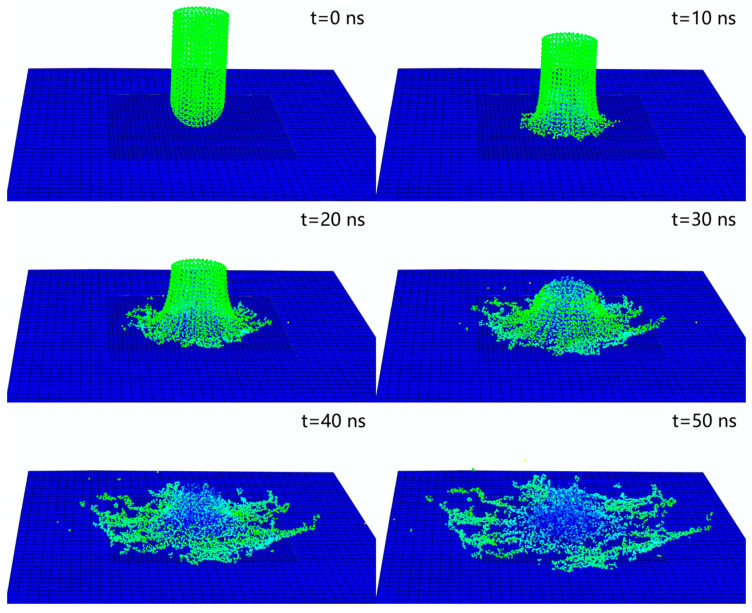
Impact figures of different points of time.

The impact duration of a single microjet on the wall is extremely short, only tens of nanoseconds. According to the previous research results of our research group, jet particles were scattered and began to splash around laterally at *t* = 20 ns. When *t* = 50 ns, the particles had almost no impact effect on the wall. This process is shown in Figure 4. Therefore, the impact duration in the simulation analysis for the single microjet was set at 50 ns [8].

Moreover, according to our previous research results, the initial speed range of the microjet was found to be between 310 and 370 m/s in the normal operating condition of ultrasonic honing [8].

**Figure 5:**
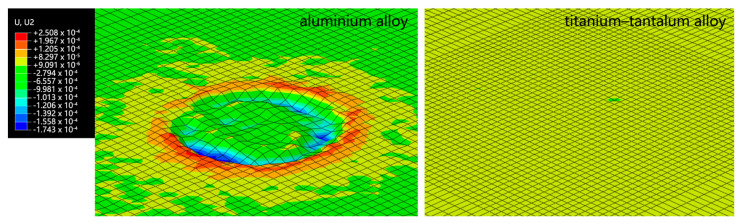
Longitudinal deformation (mm) map of alloy wall with different wall surface materials with single impact on initial speed of 310 m/s.

We first tried to set the initial speed to 310 m/s for a single impact. However, due to the high hardness of the titanium–tantalum alloy, the single impact at 310 m/s did not have an obvious effect on the wall, with a maximum depth of only 2.45 × 10^−7^ μm. Such an impact hardly produced any visible deformation. In contrast, when the wall material was the 1060 aluminum alloy, the pit depth caused by the single impact with the same initial speed was 0.17 μm, as shown in Figure 5.

We know that the pit depth is mainly affected by the initial speed and impact angle [8]. And the initial speed is the main influencing factor, so its change has a greater impact on the pit depth than the impact angle’s change. Therefore, if we want to improve the microjet’s impact effect, there are two methods to consider: the initial speed of the microjet should be increased, or the impact duration should be extended by generating repeated impacts. 

### 5.2. Single Impact Analysis at Different Initial Speeds

**Figure 6:**
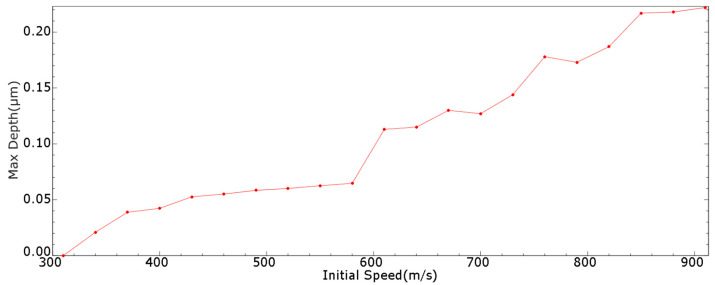
Maximum pit depth given by single impact with different initial speeds.

This section examines the effect of increasing the initial speed of the microjet. The speed was increased by 30 m/s each time from 310 m/s, and the maximum pit depth of a single impact with each initial speed was recorded, as shown in Figure 6 and Table A1 (in Appendix A).

According to the measurement accuracy of the instrument in our research group, it was believed that an obvious impact effect corresponded to a maximum pit depth of at least 1 μm. It was found that the maximum depth increased with the initial speed. When the initial speed increased from 580 to 610 m/s, the maximum depth increased from 0.0647 to 0.113 μm.

Therefore, to make an obvious impact effect using the single microjet, the initial speed must reach at least 580 to 610 m/s. The required initial speed for a maximum depth of 1.5 μm was found to be 730 to 760 m/s, and that for 2 μm was found to be 820 to 850 m/s.

**Figure 7:**
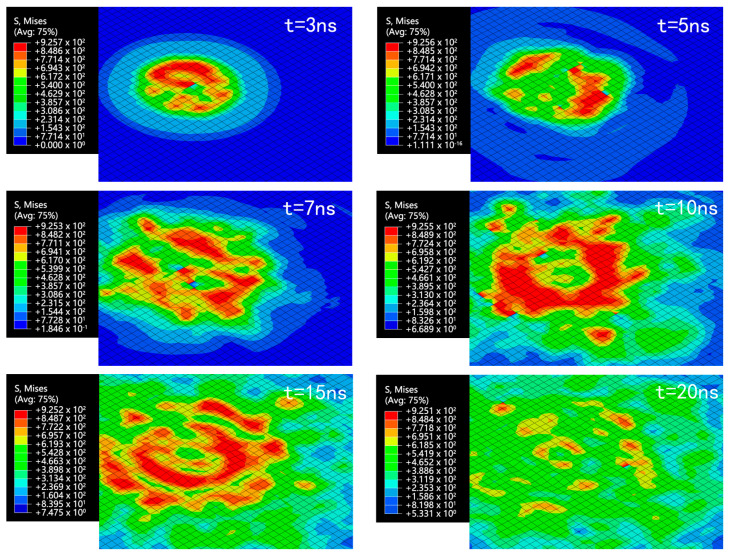
Equivalent stress (MPa) map of alloy wall.

**Figure 8:**
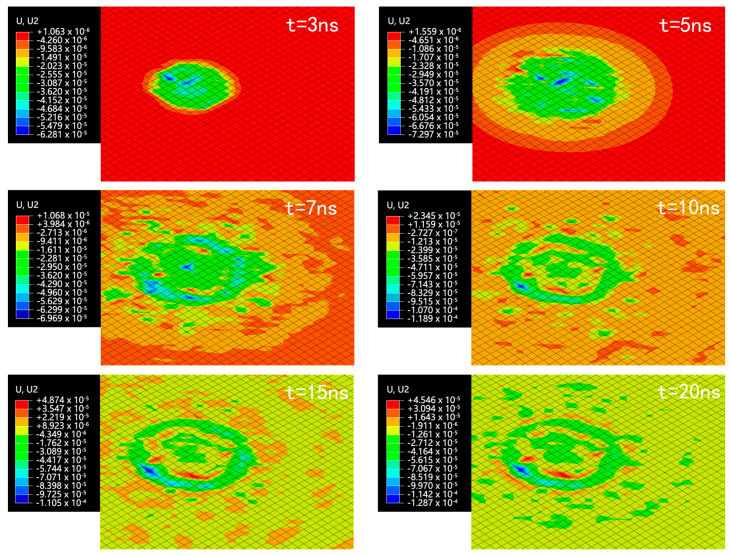
Longitudinal deformation (mm) map of alloy wall.

The alloy wall condition was analyzed with the initial speed of 610 m/s. The equivalent stress distribution at different time points is shown in Figure 7. The deformation map is shown in Figure 8.

It is observed that the area affected by the equivalent stress on the alloy wall continued to increase as the microjet impacted the wall. The equivalent stress gradually increased and stabilized at approximately 925 MPa, reaching the yield strength of Ti30Ta. This fact indicates that the microjet impact caused the plastic deformation of Ti30Ta and changed its surface topography.

The microjet impact caused wall surface deformation. From time *t* = 5 to *t* = 7 ns, stable micro-pits were formed on the wall, accompanied by material bulges at the edges of the micro-pits. These bulges were caused by the plastic deformation of the material flowing outward due to the rapid impact of the microjet. The wall deformation mainly occurred from *t* = 5 to *t* = 20 ns, with a fast increase in deformation from *t* = 5 to *t* = 15 ns and a slower increase after *t* = 15 ns. When *t* = 26 ns, the maximum equivalent stress of the wall decreased to approximately 900 MPa, followed by a continuous reduction. After *t* = 26 ns, there was almost no more deformation, and the final pit depth was approximately 0.1125 μm.

### 5.3. Continuous Impact Analysis with Different Numbers of Impacts

The initial speed of a microjet generated by an ultrasonic honing system is restricted by the system’s structure and working condition [18]. The initial speed used in this study was from 310 to 370 m/s. With other cavitation sources, microjets can achieve a higher initial speed, shown in Table 2. However, in this study, it is difficult to increase the speed to more than 610 m/s. Therefore, this section examines the effect of extended impact duration and repeated impacts.

For the convenience of analysis, it was assumed that the initial speed of each microjet was unchanged, and the next impact was started after the previous microjet had completely dissipated to remove the particle interference of the previous microjet. All other model parameters were kept unchanged. With the ABAQUS software, we exported the wall part condition after the previous impact was finished, and this wall condition was used as the initial state of the model to simulate the continuous impact process for the same impact position on the alloy surface.

It should be noted that because the wall model part was replaced after each impact, the deformation map after the *N*th impact depicted the “relative change” compared to the *N* − 1th impact, rather than the relative change with respect to the initial state. Therefore, its map value does not correspond to the maximum depth and height of the impact pit. This issue limited our analysis of the deformation map. Therefore, this section mainly summarizes the final surface morphology after successive impacts.

**Figure 9:**
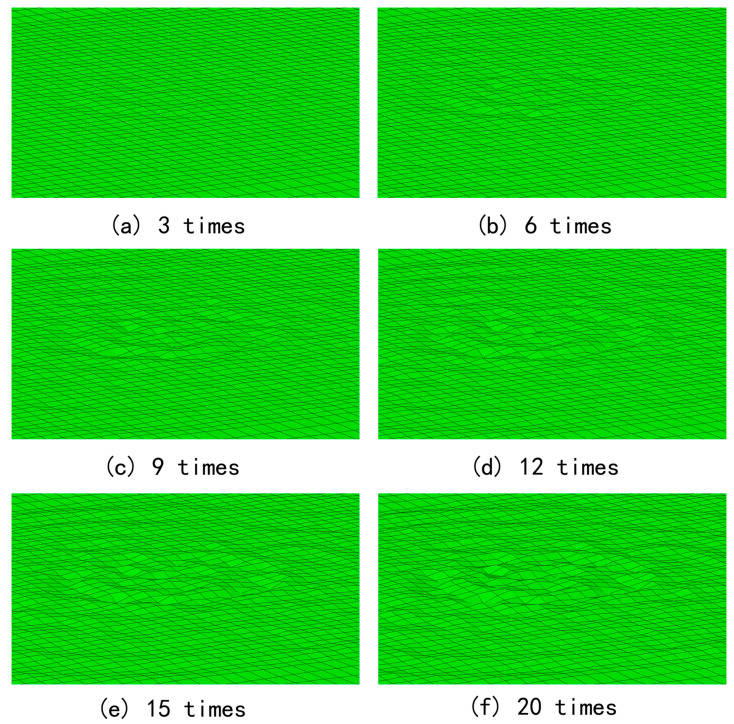
Surface morphology of alloy wall with different numbers of impacts with initial speed of 330 m/s.

To analyze the maximum pit depth, we used the HyperMesh 2021 to extract the outer surface of the model to import the data into MATLAB 2022. Then, the lowest position of the outer surface was found to determine the maximum depth of the pit area. Then, we conducted 50 consecutive impacts simulation analyses with initial speeds of 310, 330, 350 and 370 m/s, respectively. Taking 330 m/s and the first 20 times as an example, the final morphology of the wall under different numbers of impacts is shown in Figure 9.

The cumulative effect of the impacts can be clearly observed in Figure 7. The pit shape was initially developed at the sixth impact. The maximum pit depth gradually increased during the subsequent impacts, while the pit diameter and shape basically remained unchanged. Therefore, when the microjets continuously impacted the same location, the cumulative effect generated obvious pits. The pit shape was similar to that created by a single impact, with the maximum depth appearing at the edge rather than at the center and the bulge appearing at the edge.

**Figure 10:**
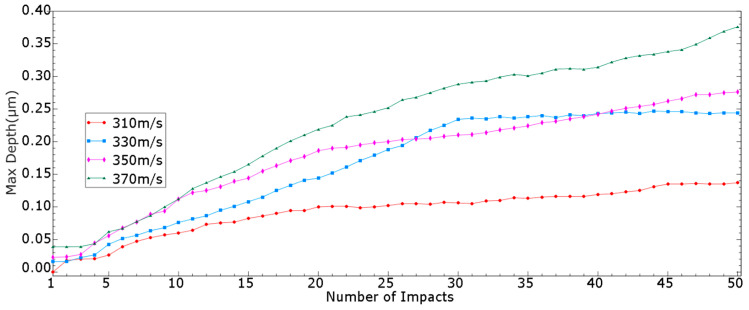
Maximum pit depth with different initial speeds and numbers of impacts.

Figure 10 and Table A2 (in Appendix A) show the pit depths formed by the first 50 consecutive impacts with initial speeds of 310, 330, 350 and 370 m/s.

It can be observed that the maximum pit depth was normally positively correlated with the initial speed and the number of impacts. At an initial speed of 310 m/s, the maximum depth reached 0.1 μm with an obvious impact effect at the 20th impact. As the initial speed further increased, the number of impacts required to reach a 0.1 μm depth decreased, from 14th to 10th and 9th, as shown in Figure 10. Normally, a higher initial speed gave a greater maximum depth when the number of impacts was kept the same.

It is noticed that there is no linear relationship between the maximum pit depth and the number of impacts. With the increase in the number of impacts, the maximum pit depth shows stagnation and negative growth rates at some times (e.g., the 31th to 50th impacts at an initial speed of 330 m/s). This may be because when the dented part of the deformed wall is impacted, the particles in this part are equivalent to oblique impact. According to the previous research of our research group [8], the oblique impact will produce a poor impact effect at a larger angle, so the impact effect of the particles in this part is not as good as that of other particles, resulting in no further deepening of the dented part.

It is also observed that the growth rate’s stagnation and negative growth only occur with 310, 330 and 350 m/s initial speed during the first 50 consecutive impacts, while the growth rate with 370 m/s initial speed is still stable. To confirm the universality of the growth pattern, we performed more analyses with a 370 m/s initial speed. The results shown in Table A3 (in Appendix A) confirm that stagnation and negative growth rates are applicable to all different initial speeds from 310 to 370 m/s.

## 6. Conclusions

The SPH–FEM coupled method was developed by treating the microjet as SPH particles and treating the wall surface as a finite element. ABAQUS was used to conduct simulation analysis for the impact process of ultrasonic cavitation microjets. The effects of initial speed and number of impacts on wall stress and deformation distributions were analyzed. The main conclusions drawn are as follows:

1. Microjet impact can cause plastic deformation on the Ti30Ta alloy surface, resulting in changes in surface topography. As the initial speed of the microjet increases, the maximum pit depth increases. The initial speeds required to produce the maximum depths of 0.1, 0.15 and 0.2 μm were 580–610, 730–760 and 820–850 m/s, respectively, with the single impact of the microjet.
2. Even if the initial speed is low, the cumulative effect formed by continuous impacts can still cause changes in the surface topography of the Ti30Ta alloy. The pit shape caused by continuous impacts is similar to that caused by a single impact. The maximum pit depth appears at the edge rather than at the center, and bulges appear at the edge.
3. The maximum pit depth increases with the number of impacts. At an initial speed of 310 m/s, the maximum depth reached 0.1 μm at the 20th impact. As the initial speed increased, the number of impacts required to reach a depth of 0.1 μm decreased. Specifically, the numbers of impacts required were 14, 12, 10 and 9 for initial speeds of 325, 340, 355 and 370 m/s, respectively.
4. When the number of impacts is large, with the increase in the number of impacts, the growth rate of the maximum pit depth gradually slows down and even shows no growth or negative growth at some times. From this perspective, after the impact has been carried out for a period of time, simply continuing to extend the impact duration is not a good way to enhance the impact effect.
5. The microjet impact caused by cavitation in ultrasonic honing can cause elastic–plastic deformation of the workpiece wall. Although the high hardness of the titanium–tantalum alloy minimizes the plastic deformation caused by a single impact, during ultrasonic honing, a large number of microjets generate continuous and repeated impacts at one position by extending the impact duration, causing non-negligible cumulative impact effects. These cumulative impacts and the accompanying shear effects can cause material loss.

## Figures and Tables

**Table 1:** Material parameters.

	Water	Ti30Ta
Density (kg·m^−3^)	1000	5773
Sound velocity (m·s^−1^)	1500	5700
Young’s modulus (MPa)		72,000
Yield strength (MPa)		925
Poisson’s ratio		0.32

**Table 2:** Microjet’s initial speed in different cavitation sources.

Thesis	Cavitation Source	Initial Speed (m·s^−1^)
[19]	Hydrodynamic and spark induction	112~500
[20]	Ultrasonic induction	192~755
[21]	Ultrasonic induction	200~700
[22]	Laser induction	300~780
[23]	Electrode induction	200~400
[24]	Electromagnetic induction	250~300

## Data Availability

Data are contained within the article.

## Appendix A

**Table A1:** Maximum pit depth given by single impact with different initial speeds.

Initial Speed (m·s^−1^)	Maximum Pit Depth (µm)
310	2.45 × 10^−7^
340	2.09 × 10^−2^
370	3.88 × 10^−2^
400	4.23 × 10^−2^
430	5.26 × 10^−2^
460	5.51 × 10^−2^
490	5.84 × 10^−2^
520	6.01 × 10^−2^
550	6.25 × 10^−2^
580	6.47 × 10^−2^
610	1.13 × 10^−1^
640	1.15 × 10^−1^
670	1.30 × 10^−1^
700	1.27 × 10^−1^
730	1.44 × 10^−1^
760	1.78 × 10^−1^
790	1.73 × 10^−1^
820	1.87 × 10^−1^
850	2.17 × 10^−1^
880	2.18 × 10^−1^
910	2.22 × 10^−1^

**Table A2:** Maximum pit depth with different initial speeds and numbers of impacts (the first 50).

Initial Speed (m·s^−1^)	Number of Impacts	Maximum Pit Depth (µm)
310	1	2.45 × 10^−7^
	2	1.79 × 10^−2^
	3	2.01 × 10^−2^
	4	2.04 × 10^−2^
	5	2.61 × 10^−2^
	6	3.92 × 10^−2^
	7	4.71 × 10^−2^
	8	5.29 × 10^−2^
	9	5.70 × 10^−2^
	10	6.00 × 10^−2^
	11	6.42 × 10^−2^
	12	7.32 × 10^−2^
	13	7.56 × 10^−2^
	14	7.67 × 10^−2^
	15	8.23 × 10^−2^
	16	8.61 × 10^−2^
	17	8.99 × 10^−2^
	18	9.42 × 10^−2^
	19	9.43 × 10^−2^
	20	1.00 × 10^−1^
	21	1.01 × 10^−1^
	22	1.01 × 10^−1^
	23	9.87 × 10^−2^
	24	1.00 × 10^−1^
	25	1.02 × 10^−1^
	26	1.05 × 10^−1^
	27	1.05 × 10^−1^
	28	1.04 × 10^−1^
	29	1.07 × 10^−1^
	30	1.06 × 10^−1^
	31	1.05 × 10^−1^
	32	1.09 × 10^−1^
	33	1.10 × 10^−1^
	34	1.14 × 10^−1^
	35	1.13 × 10^−1^
	36	1.15 × 10^−1^
	37	1.16 × 10^−1^
	38	1.16 × 10^−1^
	39	1.16 × 10^−1^
	40	1.19 × 10^−1^
	41	1.20 × 10^−1^
	42	1.23 × 10^−1^
	43	1.25 × 10^−1^
	44	1.31 × 10^−1^
	45	1.35 × 10^−1^
	46	1.35 × 10^−1^
	47	1.36 × 10^−1^
	48	1.35 × 10^−1^
	49	1.35 × 10^−1^
	50	1.37 × 10^−1^
330	1	1.61 × 10^−2^
	2	1.62 × 10^−2^
	3	2.23 × 10^−2^
	4	2.65 × 10^−2^
	5	4.26 × 10^−2^
	6	5.17 × 10^−2^
	7	5.64 × 10^−2^
	8	6.36 × 10^−2^
	9	6.81 × 10^−2^
	10	7.61 × 10^−2^
	11	8.15 × 10^−2^
	12	8.63 × 10^−2^
	13	9.47 × 10^−2^
	14	1.01 × 10^−1^
	15	1.08 × 10^−1^
	16	1.15 × 10^−1^
	17	1.25 × 10^−1^
	18	1.33 × 10^−1^
	19	1.41 × 10^−1^
	20	1.44 × 10^−1^
	21	1.52 × 10^−1^
	22	1.61 × 10^−1^
	23	1.71 × 10^−1^
	24	1.79 × 10^−1^
	25	1.88 × 10^−1^
	26	1.94 × 10^−1^
	27	2.06 × 10^−1^
	28	2.17 × 10^−1^
	29	2.25 × 10^−1^
	30	2.34 × 10^−1^
	31	2.36 × 10^−1^
	32	2.35 × 10^−1^
	33	2.38 × 10^−1^
	34	2.36 × 10^−1^
	35	2.38 × 10^−1^
	36	2.40 × 10^−1^
	37	2.37 × 10^−1^
	38	2.41 × 10^−1^
	39	2.40 × 10^−1^
	40	2.43 × 10^−1^
	41	2.44 × 10^−1^
	42	2.45 × 10^−1^
	43	2.43 × 10^−1^
	44	2.47 × 10^−1^
	45	2.46 × 10^−1^
	46	2.46 × 10^−1^
	47	2.44 × 10^−1^
	48	2.43 × 10^−1^
	49	2.44 × 10^−1^
	50	2.44 × 10^−1^
350	1	2.28 × 10^−1^
	2	2.33 × 10^−1^
	3	2.72 × 10^−1^
	4	4.43 × 10^−1^
	5	5.59 × 10^−1^
	6	6.78 × 10^−1^
	7	7.67 × 10^−1^
	8	8.87 × 10^−1^
	9	9.36 × 10^−1^
	10	1.12 × 10^−1^
	11	1.22 × 10^−1^
	12	1.25 × 10^−1^
	13	1.31 × 10^−1^
	14	1.39 × 10^−1^
	15	1.44 × 10^−1^
	16	1.55 × 10^−1^
	17	1.63 × 10^−1^
	18	1.71 × 10^−1^
	19	1.77 × 10^−1^
	20	1.86 × 10^−1^
	21	1.90 × 10^−1^
	22	1.91 × 10^−1^
	23	1.95 × 10^−1^
	24	1.98 × 10^−1^
	25	2.00 × 10^−1^
	26	2.03 × 10^−1^
	27	2.04 × 10^−1^
	28	2.05 × 10^−1^
	29	2.08 × 10^−1^
	30	2.10 × 10^−1^
	31	2.11 × 10^−1^
	32	2.14 × 10^−1^
	33	2.18 × 10^−1^
	34	2.21 × 10^−1^
	35	2.24 × 10^−1^
	36	2.29 × 10^−1^
	37	2.31 × 10^−1^
	38	2.35 × 10^−1^
	39	2.38 × 10^−1^
	40	2.42 × 10^−1^
	41	2.47 × 10^−1^
	42	2.51 × 10^−1^
	43	2.54 × 10^−1^
	44	2.57 × 10^−1^
	45	2.62 × 10^−1^
	46	2.66 × 10^−1^
	47	2.72 × 10^−1^
	48	2.72 × 10^−1^
	49	2.75 × 10^−1^
	50	2.76 × 10^−1^
370	1	3.87 × 10^−2^
	2	3.89 × 10^−2^
	3	3.92 × 10^−2^
	4	4.36 × 10^−2^
	5	6.18 × 10^−2^
	6	6.69 × 10^−2^
	7	7.74 × 10^−2^
	8	8.65 × 10^−2^
	9	1.00 × 10^−1^
	10	1.12 × 10^−1^
	11	1.28 × 10^−1^
	12	1.37 × 10^−1^
	13	1.46 × 10^−1^
	14	1.54 × 10^−1^
	15	1.65 × 10^−1^
	16	1.78 × 10^−1^
	17	1.90 × 10^−1^
	18	2.01 × 10^−1^
	19	2.10 × 10^−1^
	20	2.19 × 10^−1^
	21	2.25 × 10^−1^
	22	2.38 × 10^−1^
	23	2.41 × 10^−1^
	24	2.46 × 10^−1^
	25	2.52 × 10^−1^
	26	2.64 × 10^−1^
	27	2.68 × 10^−1^
	28	2.75 × 10^−1^
	29	2.82 × 10^−1^
	30	2.88 × 10^−1^
	31	2.91 × 10^−1^
	32	2.93 × 10^−1^
	33	2.99 × 10^−1^
	34	3.03 × 10^−1^
	35	3.01 × 10^−1^
	36	3.05 × 10^−1^
	37	3.11 × 10^−1^
	38	3.12 × 10^−1^
	39	3.11 × 10^−1^
	40	3.14 × 10^−1^
	41	3.22 × 10^−1^
	42	3.28 × 10^−1^
	43	3.32 × 10^−1^
	44	3.34 × 10^−1^
	45	3.38 × 10^−1^
	46	3.41 × 10^−1^
	47	3.49 × 10^−1^
	48	3.59 × 10^−1^
	49	3.69 × 10^−1^
	50	3.76 × 10^−1^

**Table A3:** Maximum pit depth with different numbers of impacts (after 50).

Initial Speed (m·s^−1^)	Number of Impacts	Maximum Pit Depth (µm)
370	51	3.81 × 10^−1^
	52	3.86 × 10^−1^
	53	3.91 × 10^−1^
	54	3.93 × 10^−1^
	55	3.98 × 10^−1^
	56	4.02 × 10^−1^
	57	4.07 × 10^−1^
	58	4.10 × 10^−1^
	59	4.16 × 10^−1^
	60	4.18 × 10^−1^
	61	4.20 × 10^−1^
	62	4.23 × 10^−1^
	63	4.26 × 10^−1^
	64	4.32 × 10^−1^
	65	4.43 × 10^−1^
	66	4.51 × 10^−1^
	67	4.53 × 10^−1^
	68	4.52 × 10^−1^
	69	4.54 × 10^−1^
	70	4.57 × 10^−1^
